# Metabolic reprogramming and therapeutic targeting in non-small cell lung cancer: emerging insights beyond the Warburg effect

**DOI:** 10.3389/fonc.2025.1564226

**Published:** 2025-05-21

**Authors:** Hong Cai, Feng Zhang, Fang Xu, Chunhui Yang

**Affiliations:** ^1^ Department of Clinical Laboratory, The Second Hospital of Dalian Medical University, Dalian, Liaoning, China; ^2^ Department of Clinical Laboratory, Affiliated Zhongshan Hospital of Dalian University, Dalian, Liaoning, China

**Keywords:** non-small cell lung cancer, metabolic reprogramming, tumor microenvironment, therapeutic targeting, metabolic vulnerabilities

## Abstract

Non-small cell lung cancer (NSCLC) remains a leading cause of cancer-related mortality worldwide. Recent advancements have illuminated the intricate metabolic reprogramming that underpins NSCLC progression and resistance to therapy. Beyond the classical Warburg effect, emerging evidence highlights the pivotal roles of altered lipid metabolism, amino acid utilization, and the metabolic crosstalk within the tumor microenvironment (TME). This review delves into the latest discoveries in NSCLC metabolism, emphasizing novel pathways and mechanisms that contribute to tumor growth and survival. We critically assess the interplay between cancer cell metabolism and the TME, explore the impact of metabolic heterogeneity, and discuss how metabolic adaptations confer therapeutic resistance. By integrating insights from cutting-edge technologies such as single-cell metabolomics and spatial metabolomics, we identify potential metabolic vulnerabilities in NSCLC. Finally, we propose innovative therapeutic strategies that target these metabolic dependencies, including combination approaches that enhance the efficacy of existing treatments and pave the way for personalized metabolic therapies.

## 1 Introduction

Lung cancer remains a global health challenge, being the leading cause of cancer-related mortality worldwide, with an estimated 2.2 million new cases and 1.8 million deaths in 2020 (1). NSCLC accounts for approximately 85% of all lung cancer cases and includes various histological subtypes such as adenocarcinoma, squamous cell carcinoma, and large cell carcinoma (2). Despite significant advances in surgical techniques, chemotherapy, targeted therapies, and immunotherapy, the overall five-year survival rate for NSCLC patients remains low, particularly in advanced stages where it drops below 10% (3). Late diagnosis, tumor heterogeneity, and the development of resistance to conventional treatments contribute to this poor prognosis (4). Therefore, a deeper understanding of the underlying mechanisms driving NSCLC progression and therapeutic resistance is crucial for developing more effective treatments.

One area of intense research is the metabolic reprogramming of cancer cells-a hallmark of cancer that supports rapid proliferation and survival under hostile conditions (5). Metabolic alterations enable cancer cells to meet the increased demands for energy and biosynthetic precursors required for continuous growth and division (6). Warburg effect, characterized by increased glycolysis even in the presence of oxygen, has been a focal point of cancer metabolism research (7), recent studies have uncovered a more complex metabolic landscape in NSCLC. Alterations in lipid metabolism, amino acid utilization, and metabolic interactions with the TME play significant roles in tumor progression, metastasis, and therapeutic resistance (8). Furthermore, metabolic heterogeneity within tumors and the metabolic plasticity of cancer cells allow them to adapt to changing environmental conditions and therapeutic pressures (9).

The TME, comprising stromal cells, immune cells, extracellular matrix (ECM) components, and vasculature, interacts dynamically with cancer cells, influencing their metabolic behavior and contributing to disease progression (10). Cancer-associated fibroblasts (CAFs) can alter the availability of nutrients and secrete metabolic intermediates that fuel tumor growth (11). Immune cells within the TME can have their function modulated by the metabolic activities of cancer cells, leading to immune evasion (12). Hypoxia, a common feature of solid tumors due to abnormal vasculature, further drives metabolic reprogramming by stabilizing hypoxia-inducible factors (HIFs) that regulate genes involved in glycolysis and angiogenesis (13).

This review aims to provide a comprehensive and up-to-date analysis of energy metabolism in NSCLC, highlighting novel insights and potential therapeutic opportunities. We focus on recent discoveries that shed light on the metabolic heterogeneity of NSCLC, the influence of the TME on metabolic adaptations, and the implications for therapy resistance. By integrating emerging technologies such as single-cell metabolomics, CRISPR-based metabolic screens, and systems biology approaches, and by adopting multidisciplinary perspectives, we propose innovative strategies to target metabolic vulnerabilities in NSCLC. Ultimately, we aim to bridge the gap between basic metabolic research and clinical applications, paving the way for more effective and personalized therapies for NSCLC patients.

## 2 Metabolic reprogramming in NSCLC: beyond the Warburg effect

### 2.1 The Warburg effect revisited

The Warburg effect, first described by Otto Warburg in the 1920s, refers to the observation that cancer cells preferentially utilize glycolysis for energy production even in the presence of adequate oxygen-a phenomenon known as aerobic glycolysis. This metabolic reprogramming allows cancer cells to rapidly generate ATP and accumulate intermediates for biosynthetic processes essential for proliferation (14). In NSCLC, there is significant upregulation of glycolytic enzymes such as hexokinase 2 (HK2) and pyruvate kinase M2 (PKM2), which facilitate increased glycolytic flux (15). However, this glycolytic shift is only part of the complex metabolic adaptations in NSCLC. Recent studies have revealed that mitochondrial oxidative phosphorylation (OXPHOS) remains active in many cancer cells, including NSCLC (16). This suggests that cancer cells exhibit metabolic flexibility, capable of utilizing both glycolysis and OXPHOS depending on environmental conditions and cellular demands (17). For instance, under hypoxic conditions commonly found within tumors, glycolysis is upregulated, while in oxygen-rich areas, OXPHOS can contribute significantly to adenosine triphosphate (ATP) production (18).

Moreover, the reliance on glycolysis is influenced by oncogenic signaling pathways. Mutations in genes such as kirsten rats arcomaviral oncogene homolog (KRAS) and epidermal growth factor receptor (EGFR), which are prevalent in NSCLC, activate downstream effectors like phosphatidylinositol 3-Kinase/protein kinase B/mammalian target of rapamycin (PI3K/AKT/mTOR) and mitogen-activated protein kinase (MAPK) pathways (19). These pathways upregulate glucose transporters (e.g., GLUT1) and glycolytic enzymes, enhancing glucose uptake and glycolysis (20). Additionally, HIFs, stabilized under low oxygen conditions, promote the expression of genes involved in glycolysis and suppress OXPHOS (21).

Understanding the nuances of the Warburg effect in NSCLC is crucial for therapeutic development. Targeting glycolytic enzymes has shown promise in preclinical models; however, due to the metabolic plasticity of cancer cells, inhibition of glycolysis alone may lead to compensatory upregulation of OXPHOS or other pathways. Therefore, combination therapies that target multiple metabolic pathways may be more effective in overcoming resistance and achieving sustained antitumor effects.

### 2.2 Mitochondrial metabolism and OXPHOS

**Figure 1:**
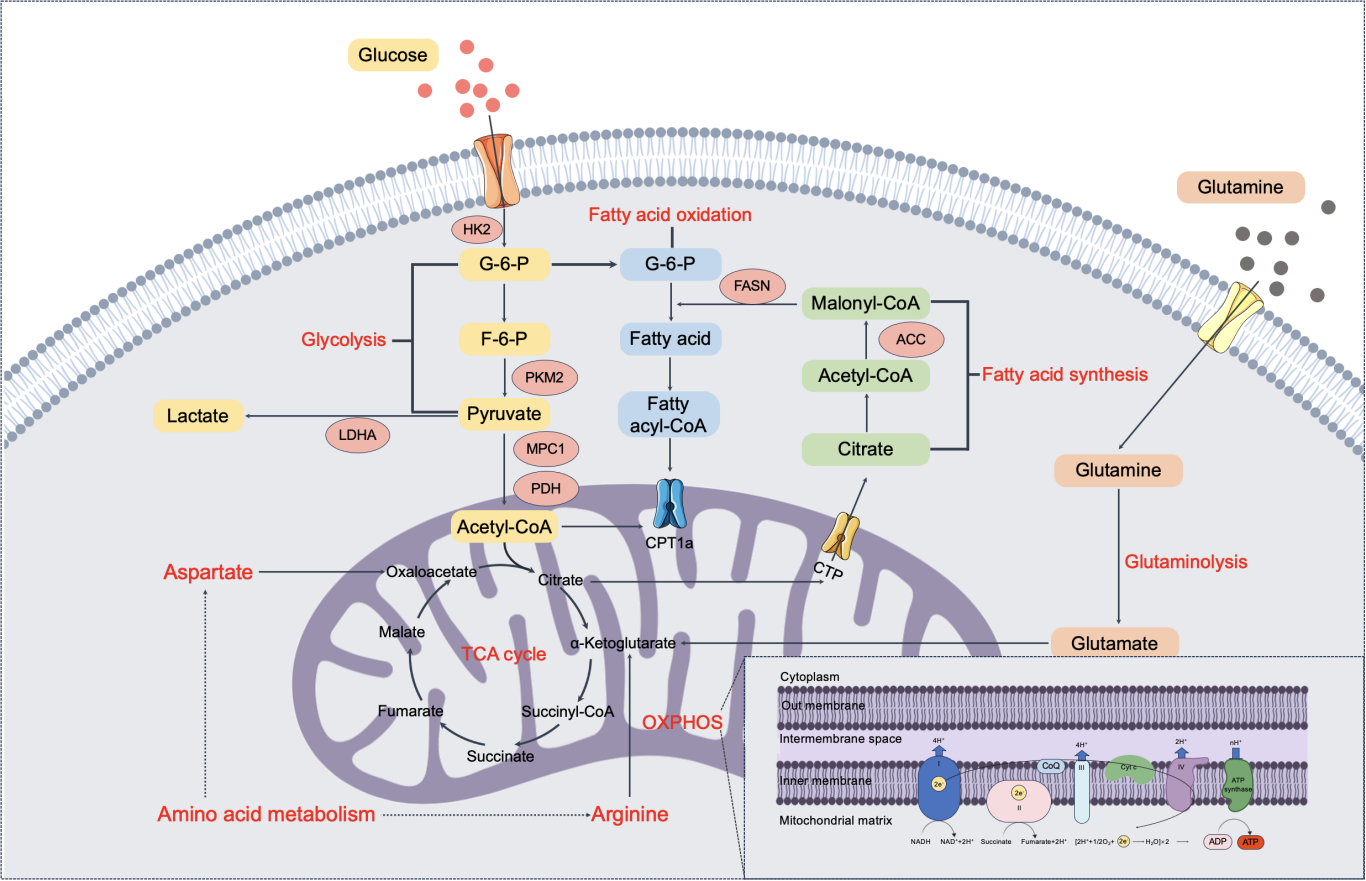
Integrated schematic of glycolysis, mitochondrial metabolism, and lipid pathways in NSCLC cells. This figure summarizes key metabolic processes in NSCLC cells, illustrating the interplay and compensatory mechanisms between glycolysis and OXPHOS, as well as the integration of lipid metabolism. Glucose is metabolized through glycolysis to produce pyruvate, which enters mitochondria to fuel the TCA cycle and OXPHOS for ATP production. Under certain conditions, pyruvate is diverted to lactate via LDHA. Glutamine contributes to TCA cycle intermediates through glutaminolysis, supporting biosynthesis and redox balance. Fatty acid metabolism is also reprogrammed in NSCLC: fatty acid synthesis, driven by enzymes such as ACC and FASN, converts citrate and acetyl-CoA into lipids, while FAO via CPT1a provides additional acetyl-CoA for mitochondrial respiration. Together, these interconnected pathways reflect the metabolic flexibility and adaptability of NSCLC cells in response to environmental and therapeutic pressures.

Contrary to the traditional view that cancer cells have impaired mitochondrial function, recent studies have demonstrated that mitochondrial OXPHOS remains active and is essential for the survival and proliferation of NSCLC cells (22). Mitochondria play a pivotal role not only in energy production but also in biosynthesis, redox balance, and regulation of apoptosis (23). NSCLC cells exhibit remarkable metabolic flexibility, enabling them to switch between glycolysis and OXPHOS in response to environmental cues such as nutrient availability, oxygen levels, and therapeutic interventions (24). **Figure 1** illustrates the dynamic interplay between these two energy-producing pathways and highlights the compensatory mechanisms that allow NSCLC cells to adapt their metabolism under stress conditions.

The metabolic adaptability contributes significantly to therapeutic resistance. For instance, when glycolysis is inhibited-either pharmacologically or due to nutrient scarcity-NSCLC cells can upregulate OXPHOS to meet their energy and biosynthetic demands (25). This compensatory increase in OXPHOS allows cancer cells to evade glycolysis-targeted therapies, highlighting the challenge of metabolic plasticity in effective cancer treatment (26). Additionally, some subpopulations of cancer stem cells within NSCLC have been found to rely heavily on OXPHOS, contributing to tumor heterogeneity and resistance to chemotherapy and radiotherapy (27).

Recent research has uncovered that oncogenic drivers common in NSCLC, such as mutations in the KRAS gene, can influence mitochondrial function. KRAS-mutant NSCLC cells demonstrate enhanced mitochondrial biogenesis and elevated OXPHOS activity, which supports their aggressive phenotype (28). Moreover, alterations in mitochondrial dynamics-processes that control mitochondrial fission and fusion-have been implicated in NSCLC progression. Dysregulated expression of proteins like dynamin-related protein 1 (DRP1) and mitofusins (MFN1 and MFN2) affects mitochondrial morphology and function, promoting cancer cell survival and metastasis (29). Targeting mitochondrial metabolism presents a promising therapeutic strategy. Inhibitors of OXPHOS components, such as complex I inhibitor IACS-010759, have shown antitumor activity in preclinical models of NSCLC by inducing energy stress and apoptosis (30). Furthermore, combining OXPHOS inhibitors with agents targeting glycolysis may overcome metabolic compensation mechanisms and enhance therapeutic efficacy (31). Agents that disrupt mitochondrial dynamics or promote mitochondrial dysfunction are also being explored as potential treatments (32).

The interplay between mitochondrial metabolism and the TME further complicates the metabolic landscape. Hypoxic regions within tumors can influence mitochondrial function and promote metabolic reprogramming (33). Additionally, interactions with stromal cells and immune cells can modulate mitochondrial activity in NSCLC cells, affecting tumor growth and response to therapy (34).

In summary, mitochondrial metabolism and OXPHOS play critical roles in NSCLC biology. Understanding the mechanisms underlying metabolic flexibility and mitochondrial function in cancer cells is essential for developing effective therapeutic strategies that can circumvent resistance and target the metabolic vulnerabilities of NSCLC.

### 2.3 Lipid metabolism: a new frontier

Lipid metabolism has emerged as a critical aspect of NSCLC biology, influencing tumor growth, survival, and metastasis (35). Cancer cells require a continuous supply of lipids for the synthesis of cellular membranes and signaling molecules that support rapid proliferation. Enhanced *de novo* lipogenesis—the endogenous production of fatty acids from non-lipid precursors like glucose and glutamine—is a hallmark of metabolic reprogramming in cancer (36), and is particularly prominent in NSCLC.

This process is depicted in **Figure 1**, which illustrates key pathways involved in lipid synthesis and oxidation in NSCLC cells. This process provides not only structural components for membrane biogenesis but also lipid signaling molecules that can activate oncogenic pathways. Enzymes such as fatty acid synthase (FASN) and stearoyl-CoA desaturase-1 (SCD1) play pivotal roles in lipid synthesis and are overexpressed in NSCLC (37). FASN is responsible for the synthesis of palmitate, a saturated fatty acid that serves as a building block for more complex lipids. Overexpression of FASN has been associated with increased tumor aggressiveness, resistance to chemotherapy, and poorer prognosis in NSCLC patients (38). Targeting FASN with small-molecule inhibitors has shown promise in preclinical models, leading to reduced tumor growth and enhanced sensitivity to other therapies (39). SCD1 introduces a double bond into saturated fatty acyl-CoAs to produce MUFAs, which are essential for maintaining membrane fluidity and function. Elevated SCD1 expression has been linked to enhanced tumor growth, metastasis, and reduced survival rates in NSCLC. Inhibition of SCD1 can disrupt membrane composition, induce endoplasmic reticulum (ER) stress, and trigger apoptosis in cancer cells (40). Acetyl-CoA carboxylase (ACC) is a key rate-limiting enzyme in fatty acid synthesis, playing a critical role in cellular metabolism and tumor growth by converting acetyl-CoA to malonyl-CoA. ACC regulates *de novo* fatty acid synthesis to meet the biosynthetic demands of tumor growth, and its inhibitor ND-646 significantly suppresses the growth and viability of NSCLC cells while enhancing the efficacy of chemotherapy, making it a potential target for cancer metabolism therapy (41).

Beyond lipid synthesis, NSCLC cells can utilize lipid oxidation through fatty acid oxidation (FAO) pathways to meet their energy demands and maintain redox balance (42). FAO involves the breakdown of fatty acids in mitochondria to generate acetyl-CoA, nicotinamide adenine dinucleotide (NADH), and flavin adenine dinucleotide hydrogen (FADH_2_), which feed into the tricarboxylic acid (TCA) cycle and electron transport chain to produce ATP (43). By relying on FAO, cancer cells can adapt to nutrient-deprived or hypoxic conditions where glycolysis may be less efficient (44). FAO also contributes to the maintenance of redox homeostasis by generating NADPH, a critical reducing agent that helps neutralize reactive oxygen species (ROS) and protect cells from oxidative stress (45). Enzymes like carnitine palmitoyltransferase 1 (CPT1), which regulates the transport of long-chain fatty acids into mitochondria, are often upregulated in NSCLC. Inhibition of CPT1 can impair FAO, leading to energy stress and increased sensitivity to oxidative damage (46).

NSCLC cells can enhance lipid uptake from the microenvironment by overexpressing lipid transporters such as cluster of differentiation 36 (CD36) and fatty acid-binding proteins (FABPs) (47). This uptake allows cancer cells to utilize exogenous fatty acids for energy production and membrane synthesis. Additionally, cancer cells can store excess lipids in lipid droplets, which serve as reservoirs that can be mobilized during times of metabolic stress (48). Alterations in lipid metabolism are often driven by oncogenic signaling pathways common in NSCLC. For example, activation of the PI3K/AKT/mTOR pathway can upregulate lipid synthesis by increasing the expression and activity of lipogenic enzymes (49). Mutations in KRAS, frequently observed in NSCLC, have been shown to enhance lipid metabolism, promoting tumor growth and survival (50). These signaling pathways not only stimulate lipid production but also integrate metabolic cues with cell proliferation and survival mechanisms.

### 2.4 Amino acid metabolism: beyond glutamine addiction

While glutamine metabolism is well-established in cancer biology due to its role in supporting rapid cell proliferation and survival (51), NSCLC cells also exploit other amino acids to meet their metabolic demands. Recent studies have highlighted alterations in the metabolism of amino acids such as serine, glycine, proline, and branched-chain amino acids (BCAAs), which contribute to nucleotide synthesis, redox balance, and energy production (52).

#### 2.4.1 Serine and glycine metabolism

Serine and glycine are non-essential amino acids that play crucial roles in one-carbon metabolism, which is essential for nucleotide synthesis, methylation reactions, and antioxidant defense (53). NSCLC cells can upregulate enzymes involved in the serine-glycine synthesis pathway, such as phosphoglycerate dehydrogenase (PHGDH), phosphoserine aminotransferase (PSAT1), and serine hydroxymethyltransferase (SHMT) (54). Overexpression of PHGDH has been observed in NSCLC and is associated with enhanced tumor growth and poor prognosis (55). Targeting this pathway can disrupt nucleotide biosynthesis and reduce the proliferation of cancer cells. Moreover, serine and glycine contribute to the synthesis of glutathione, a major intracellular antioxidant that helps maintain redox homeostasis (56). By elevating serine and glycine metabolism, NSCLC cells enhance their capacity to detoxify ROS, thereby promoting survival under oxidative stress conditions induced by therapies (57).

#### 2.4.2 Proline metabolism

Proline metabolism is another pathway exploited by NSCLC cells to support tumor growth and metastasis (58). Proline biosynthesis from glutamate involves the enzyme pyrroline-5-carboxylate synthase (P5CS), while its degradation is mediated by proline dehydrogenase (PRODH). Proline can serve as a source of energy and contribute to redox balance by generating NADP^+^/NADPH (59). Altered proline metabolism aids in the adaptation of cancer cells to hypoxic conditions and nutrient deprivation, facilitating tumor progression (60). Inhibiting key enzymes in proline metabolism may impair cancer cell survival and sensitize tumors to treatment (61).

#### 2.4.3 BCAAs

BCAAs-leucine, isoleucine, and valine-are essential amino acids involved in protein synthesis and signaling pathways that regulate cell growth and metabolism (62). NSCLC cells can exhibit increased uptake and catabolism of BCAAs to fuel the TCA cycle and provide nitrogen for nucleotide and amino acid synthesis (52). Enzymes such as branched-chain amino acid transaminase 1 (BCAT1) are upregulated in NSCLC and have been associated with tumor aggressiveness and poor clinical outcomes (63). Targeting BCAA metabolism may disrupt energy production and biosynthesis, leading to reduced tumor growth.

#### 2.4.4 Amino acid transporters

To support increased amino acid demands, NSCLC cells often upregulate amino acid transporters. Transporters like solute carrier family 1 member 5 (SLC1A5/ASCT2) and SLC7A5 (LAT1) facilitate the uptake of glutamine, serine, leucine, and other amino acids (64, 65). Overexpression of these transporters has been linked to enhanced tumor growth, metastasis, and resistance to chemotherapy (66). Inhibiting amino acid transporters can reduce the intracellular availability of critical nutrients, inducing metabolic stress and apoptosis in cancer cells (67).

### 2.5 Metabolic heterogeneity and plasticity

NSCLC exhibit significant metabolic heterogeneity, both between different tumors (intertumoral heterogeneity) and within individual tumors (intratumoral heterogeneity)^1^. This heterogeneity arises from a complex interplay of genetic mutations, epigenetic modifications, tumor microenvironmental factors, and cellular interactions, leading to diverse metabolic phenotypes among cancer cells (68).

#### 2.5.1 Genetic mutations and metabolic diversity

Genetic mutations commonly found in NSCLC, such as alterations in KRAS, EGFR, anaplastic lymphoma kinase (ALK), and liver kinase B1 (LKB1), drive distinct metabolic reprogramming in tumor cells (69). For instance, KRAS-mutant NSCLC cells often exhibit enhanced glucose uptake and glycolysis, whereas EGFR-mutant cells may rely more on glutamine metabolism (70). Loss of LKB1 function is associated with defects in mitochondrial OXPHOS and increased dependency on alternative energy sources (71). These genetic differences contribute to metabolic heterogeneity, influencing how tumor cells utilize nutrients and respond to metabolic stress.

#### 2.5.2 Microenvironmental influences

The TME significantly impacts metabolic heterogeneity. Factors such as hypoxia, nutrient availability, pH changes, and interactions with stromal cells create spatial metabolic gradients within tumors (72). Hypoxic regions often lead to increased glycolysis and lactate production, while well-oxygenated areas may favor OXPHOS (73). Additionally, the availability of nutrients like glucose, amino acids, and lipids can vary within the tumor, forcing cancer cells to adapt their metabolism accordingly (74).

#### 2.5.3 Interactions with stromal cells

CAFs, immune cells, and endothelial cells within the TME modulate cancer cell metabolism through paracrine signaling and direct cell-cell interactions (75). For example, CAFs can secrete metabolites such as lactate, amino acids, and fatty acids, which cancer cells uptake and utilize for energy and biosynthesis (10). Immune cells like tumor-associated macrophages (TAMs) produce cytokines that alter metabolic pathways in cancer cells, promoting survival and proliferation (76). These interactions further enhance metabolic diversity within the tumor.

#### 2.5.4 Metabolic plasticity and therapeutic resistance

Metabolic heterogeneity contributes to therapeutic resistance by enabling subpopulations of cancer cells to survive under treatment-induced stress (77). Cancer cells with different metabolic profiles may respond variably to therapies targeting specific metabolic pathways (78). Metabolic plasticity-the ability of cancer cells to switch between metabolic states-allows them to adapt to environmental changes or therapeutic pressures (79). For instance, inhibiting glycolysis may lead some cancer cells to increase OXPHOS or utilize alternative substrates like fatty acids and amino acids (80).

#### 2.5.5 Implications for personalized medicine

The presence of metabolic heterogeneity underscores the need for personalized metabolic interventions in NSCLC (81). Therapeutic strategies that consider the specific metabolic dependencies of a patient’s tumor may improve treatment efficacy (82). Techniques such as single-cell metabolomics and metabolic imaging can identify metabolic subtypes within tumors, guiding the selection of targeted therapies (83). Additionally, combining metabolic inhibitors with other treatments may overcome resistance by targeting multiple metabolic pathways simultaneously (84).

Understanding the mechanisms driving metabolic heterogeneity and plasticity is crucial for developing effective therapies. Integrating genomic, transcriptomic, and metabolomic data can provide a comprehensive view of tumor metabolism (85). Personalized approaches that tailor treatments based on individual metabolic profiles hold promise for improving outcomes in NSCLC patients.

## 3 The TME: metabolic crosstalk and therapeutic resistance

### 3.1 CAFs and metabolic support

CAFs are among the most abundant stromal cells within the TME of NSCLC and play a crucial role in supporting tumor metabolism and progression (86). CAFs undergo significant metabolic reprogramming that enables them to supply essential nutrients and metabolites to cancer cells, thereby promoting tumor growth and survival (51). One of the key phenomena illustrating this supportive role is the “reverse Warburg effect”. Unlike the traditional Warburg effect, where cancer cells preferentially utilize glycolysis for energy production even in the presence of oxygen, the reverse Warburg effect describes how CAFs enhance their glycolytic activity to produce high-energy metabolites such as lactate and pyruvate (87). These metabolites are then secreted into the TME and taken up by cancer cells, which utilize them through OXPHOS to generate ATP and support anabolic processes. This metabolic coupling allows cancer cells to conserve glucose for biosynthetic pathways, thus facilitating rapid proliferation and growth (88).

Beyond lactate production, CAFs secrete a variety of nutrients, including amino acids (e.g., glutamine, alanine) and fatty acids, which cancer cells can exploit. CAF-derived glutamine serves as an anaplerotic substrate replenishing TCA cycle intermediates in cancer cells, supporting energy production and biosynthesis of nucleotides and amino acids (89). Alanine secreted by CAFs can be converted into pyruvate, further fueling the TCA cycle. CAFs can release free fatty acids that cancer cells uptake and utilize for β-oxidation, contributing to ATP generation and membrane synthesis (90).

CAFs also modulate the ECM and secrete cytokines and growth factors that influence cancer cell metabolism and behavior (91). Factors such as transforming growth factor-beta (TGF-β), hepatocyte growth factor (HGF), and interleukins secreted by CAFs can activate signaling pathways (e.g., PI3K/AKT, MAPK) in cancer cells, leading to enhanced glycolysis and survival (92).

### 3.2 Immune cell metabolism and immune evasion

**Figure 2:**
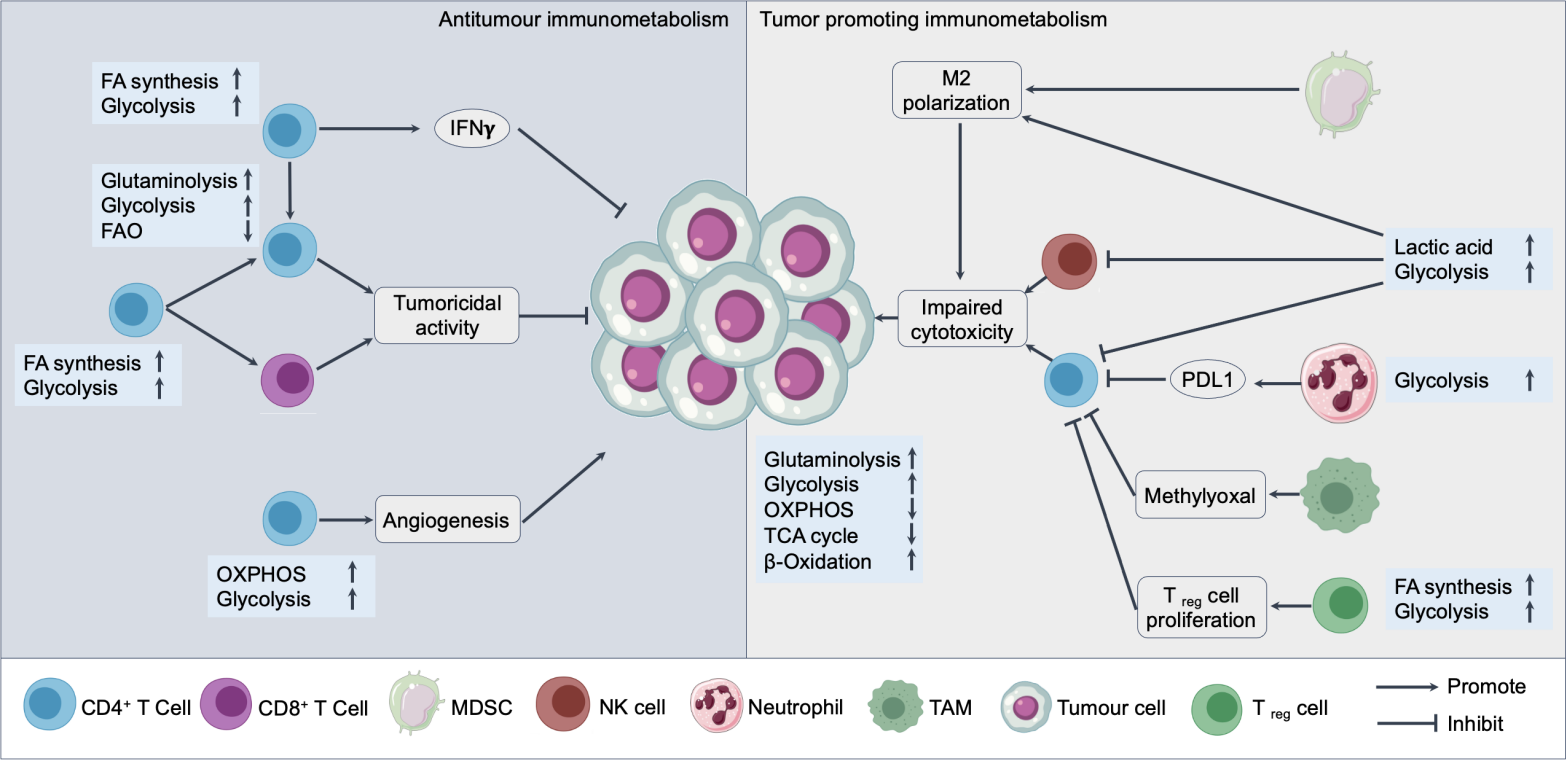
Immunometabolic interactions in the NSCLC TME.This schematic illustrates the dual roles of immune cell metabolism in either promoting or suppressing tumor growth in NSCLC. On the left, antitumor immunometabolism is driven by activated CD4^+^ and CD8^+^ T cells, NK cells, and other cytotoxic immune cells exhibiting increased glycolysis, fatty acid (FA) synthesis, OXPHOS, and glutaminolysis, supporting IFN-γ production, angiogenesis, and tumoricidal activity. On the right, tumor-promoting immunometabolism is characterized by immunosuppressive cells such as regulatory T cells (T_reg_ cell), myeloid-derived suppressor cells (MDSCs), TAMs, and neutrophils, which exploit elevated glycolysis, lactic acid production, and altered metabolic intermediates (e.g., methylglyoxal) to inhibit cytotoxic function and promote T_reg_ cell proliferation, PD-L1 expression, and M2 polarization. Tumor cells actively shape the metabolic landscape of the TME by rewiring their own metabolic programs (e.g., enhanced glycolysis, glutaminolysis, and β-oxidation) to suppress immune responses and sustain growth.

The metabolic state of immune cells within the TME profoundly affects their function and the overall immune response against NSCLC (93). Effective antitumor immunity relies on the activity of various immune cells, particularly effector T cells and natural killer (NK) cells, which require substantial energy and biosynthetic materials to proliferate and exert their cytotoxic functions (94). However, the TME is often characterized by metabolic competition and deprivation, as rapidly proliferating tumor cells consume large amounts of glucose, amino acids, and other nutrients (95). As illustrated in **Figure 2**, this metabolic imbalance not only limits nutrient availability for immune cells but also drives the reprogramming of both effector and regulatory immune cell populations, ultimately shaping the immune landscape toward either tumor suppression or immune evasion.

#### 3.2.1 Metabolic competition and nutrient deprivation

Tumor cells alter the availability of key nutrients in the TME by upregulating glucose transporters and amino acid transporters, leading to increased uptake and consumption of glucose and amino acids (96). This metabolic competition results in a nutrient-deprived environment for immune cells. Effector T cells rely on glycolysis for energy production and effector functions such as cytokine production and proliferation. Glucose deprivation impairs T cell receptor (TCR) signaling, reduces cytokine production (e.g., interferon-gamma), and diminishes cytotoxic activity (97). Amino acids like glutamine, arginine, and tryptophan are critical for T cell function and proliferation (98). Tumor cells can deplete these amino acids or produce immunosuppressive metabolites (e.g., kynurenine from tryptophan catabolism via indoleamine 2,3-dioxygenase [IDO]) that inhibit T cell activity (99).

#### 3.2.2 Metabolic checkpoints and immune suppression

Metabolic reprogramming in lung cancer plays a pivotal role in immune regulation by inducing metabolic stress and activating metabolic checkpoints in immune cells, leading to immunosuppression. Tumor-induced energy stress activates key metabolic sensors such as AMP-Activated Protein Kinase (AMPK), which modulates T cell metabolism and reduces their effector functions. Similarly, nutrient deprivation within the TME inhibits mTOR signaling, impairing T cell growth and responses. Additionally, hypoxia stabilizes HIFs, which alter immune cell metabolism and promote an immunosuppressive phenotype (100).

Tumor cells exploit these metabolic pathways to evade immune responses through several mechanisms. First, the upregulation of immune checkpoint molecules such as programmed cell death ligand 1 (PD-L1) on tumor cells interacts with programmed cell death protein 1 (PD-1) on T cells, suppressing glucose uptake and glycolysis in T cells, thereby diminishing their effector functions (101). Second, tumor cells secrete immunosuppressive factors like TGF-β and adenosine, which further modulate immune cell metabolism and suppress antitumor immunity. These metabolic interactions underscore the critical role of metabolic reprogramming in shaping the immune landscape of lung cancer, presenting potential targets for therapeutic intervention (102).

### 3.3 Hypoxia and metabolic adaptations

Hypoxia, or low oxygen conditions, is a hallmark of the TME in solid cancers, including NSCLC (103). Rapid tumor growth often outpaces the development of new blood vessels, leading to regions of insufficient oxygen supply. Hypoxic conditions trigger the stabilization of HIFs, particularly HIF-1α and HIF-2α, which are key transcription factors orchestrating cellular responses to low oxygen levels (104). Under normoxic conditions, HIF-α subunits are hydroxylated by prolyl hydroxylase domain proteins (PHDs), marking them for degradation via the von Hippel-Lindau (VHL) ubiquitin-proteasome pathway (105). Hypoxia inhibits PHD activity, preventing HIF-α degradation. Stabilized HIF-α translocates to the nucleus, dimerizes with HIF-1β, and activates the transcription of target genes involved in crucial processes such as metabolism, angiogenesis, erythropoiesis, and cell survival (106).

HIFs play a pivotal role in reprogramming cancer cell metabolism to adapt to hypoxic conditions (107). HIF-1α upregulates glycolytic enzymes, including HK2, phosphofructokinase 1 (PFK1), and lactate dehydrogenase A (LDHA), shifting the metabolic flux towards glycolysis despite the presence of oxygen (aerobic glycolysis) (108). This shift allows cancer cells to generate ATP efficiently under low oxygen conditions. Upregulation of glucose transporters such as GLUT1 enhances glucose uptake from the extracellular environment, providing substrates for glycolysis (109). HIF-1α induces pyruvate dehydrogenase kinase 1 (PDK1), which inhibits pyruvate dehydrogenase (PDH), decreasing the conversion of pyruvate to acetyl-CoA and thus reducing entry into the TCA cycle (110). This adaptation minimizes oxygen consumption and reduces ROS production from mitochondria. Increased LDHA activity converts pyruvate to lactate, which is exported out of the cell via monocarboxylate transporters (MCTs), contributing to the acidic TME (111).

## 4 Emerging technologies unveiling metabolic vulnerabilities

The rapid advancement of innovative technologies has significantly enhanced our ability to uncover metabolic vulnerabilities in cancer, providing new insights into tumor biology and therapeutic opportunities. Single-cell metabolomics has emerged as a powerful tool, enabling the analysis of metabolic heterogeneity at an unprecedented resolution (112). By profiling individual cells, researchers can identify distinct subpopulations with unique metabolic dependencies, which can inform the development of more precise and effective targeted therapies. Spatial metabolomics further expands this capability by detecting and imaging metabolites with spatial resolution at the tissue or cellular level (113). By combining mass spectrometry imaging with traditional metabolomics approaches, this technique allows for the visualization of metabolite distribution within biological tissues, offering a deeper understanding of the TME and its metabolic interactions.

The advent of high-resolution mass spectrometry (HRMS) has significantly enhanced the sensitivity and accuracy of metabolite detection. HRMS provides precise molecular weight measurements and detailed structural information, making it a cornerstone technology in metabolomics research (114). Finally, systems biology and computational modeling integrate multi-omics data to construct metabolic networks and predict therapeutic outcomes (115). Computational tools enable the simulation of metabolic interventions and identification of synergistic drug combinations, paving the way for more strategic and effective treatment regimens. Together, these emerging technologies are revolutionizing our understanding of cancer metabolism and driving the discovery of novel therapeutic strategies.

## 5 Therapeutic implications and strategies

**Figure 3:**
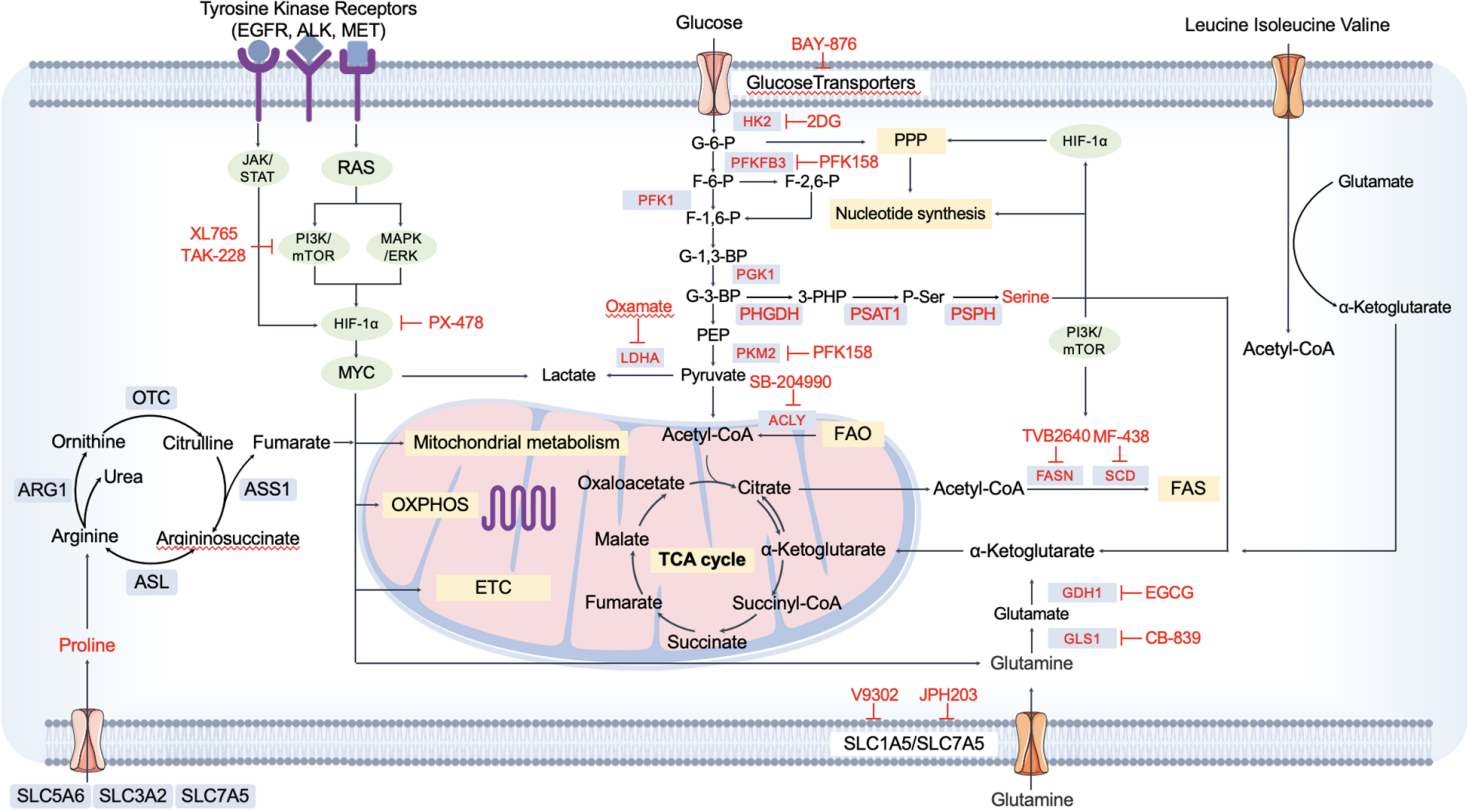
Targetable metabolic pathways and inhibitors in NSCLC.This schematic highlights key metabolic pathways reprogrammed in NSCLC cells and outlines current therapeutic targets under investigation. Central carbon metabolism—including glycolysis, the pentose phosphate pathway (PPP), TCA cycle, and OXPHOS—is regulated by oncogenic signaling pathways such as PI3K/mTOR and MAPK/ERK, often activated by mutations in receptor tyrosine kinases (EGFR, ALK, MET). Metabolic enzymes (e.g., HK2, PFKFB3, LDHA, PHGDH, PKM2, ACLY, FASN, SCD, GLS1) and transporters (e.g., SLC1A5/SLC7A5) are shown with corresponding small-molecule inhibitors (e.g., 2-DG, BAY-876, PFK158, Oxamate, PX-478, CB-839, TVB2640, MF-438, JPH203), many of which are being evaluated in preclinical or clinical settings. The diagram also illustrates amino acid metabolism (e.g., glutaminolysis, serine biosynthesis, arginine and proline metabolism), emphasizing the metabolic plasticity of NSCLC and the opportunities for combinatorial metabolic interventions. This comprehensive map provides a framework for rational design of therapies targeting metabolic vulnerabilities in NSCLC.

Recent advances in elucidating the metabolic landscape of NSCLC have highlighted several key pathways that are amenable to therapeutic intervention. Targeting metabolic dependencies represents a promising strategy to disrupt tumor growth and overcome resistance mechanisms (**Figure 3**). In this section, we discuss emerging therapeutic approaches aimed at modulating core metabolic processes in NSCLC. A summary of representative metabolic inhibitors and their targeted pathways is provided in **Table 1**, which offers a comprehensive overview of current therapeutic agents explored in NSCLC metabolic intervention.

**Table 1:** Drugs/compounds targeting different proteins/enzymes of the metabolic pathway.

Pathways	Molecular target	Drugs/compounds	Mechanism of action	Clinical trial status
*Glucose metabolism pathway*				
	GLUTS	BAY-876	selective GLUT1 inhibitor, inhibition of proliferation in NSCLC cells (20)	–
	PFK2/PFKFB3	PFK158	Inhibits growth of NSCLC (in vitro) (26)	Phase I NCT02044861
	HK2	2-DG	Specific HK2 inhibitor, induction apoptosis of lung cancer stem cells (116)	Phase I/II NCT00096707
	PKM2	TEPP-46 and DASA-58	Inhibits proliferation and induces apoptosis of NSCLC cells (117, 118)	–
	LDH-A	Oxamate	Enhances the efficacy of anti-PD-1 treatment in an NSCLC (119)	–
*Mitochondrial metabolism*				
	OXPHOS	Metformin Phenformin	Inhibit mitochondrial complex I, reducing ATP production and increasing ROS generation (120, 121)	Phase I/II NCT03086733 -
	ATP Synthase	Oligomycin	Inhibit ATP synthase (122)	–
	Bcl-2/Bcl-xL	venetoclax	Promote apoptosis (123)	Phase I NCT04274907
*Lipid metabolism pathway*				
	ACC	ND-646	Prevents ACC subunit dimerization (41)	Preclinical
	FASN	TVB2640 TVB3166	Selective FASN inhibitor (124) Selective FASN inhibitor (125)	Phase II (NCT03808558) Preclinical
	SCD1	MF-438	Specific SCD1 inhibitor, induction apoptosis lung cancer stem cells (126, 127)	Preclinical
	CPT1	Etomoxir	Irreversible inhibitor of CPT1B (46)	–
*Amino acid metabolism pathway*				
	SLC1A5	V9302	Inhibits proliferation and induces apoptosis of NSCLC cells (64)	–
	SLC7A5	JPH203	Selective SLC7A5 inhibitor (65)	–
	GLS1	CB-839	Induction apoptosis lung cancer stem cells (128)	Phase I/ II (NCT02071862) (NCT02771626)
*Signaling proteins and transcription factors*				
	mTORC1 and mTORC2	TAK-228	ATP-dependent mTOR1/2 inhibitor, inhibition of proliferation in NSCLC cells (49)	Phase II (NCT02503722)
	HIF-1α	PX-478	Induction apoptosis of NSCLC cells (104)	Preclinical
	PI3K and mTOR	XL765	A pan PI3K/mTOR inhibitor (19)	Phase I (NCT00777699)

### 5.1 Targeting glycolysis and OXPHOS

The metabolic reprogramming of NSCLC cells presents a strategic opportunity for therapeutic intervention by targeting key energy-producing pathways. Glycolysis and OXPHOS are central to cancer cell metabolism, providing ATP and metabolic intermediates necessary for rapid proliferation and survival. Inhibitors targeting these pathways have shown promise in preclinical models, but due to metabolic plasticity, cancer cells can switch between glycolysis and OXPHOS to compensate when one pathway is inhibited (129). Therefore, combination therapies targeting both pathways may be more effective in preventing metabolic compensation and inducing cancer cell death.

Key glycolytic enzymes such as HK2 and PKM2 are overexpressed in NSCLC and are critical for maintaining the high glycolytic flux observed in cancer cells. HK2 catalyzes the first step of glycolysis, phosphorylating glucose to glucose-6-phosphate. Inhibitors like 2-deoxy-D-glucose (2-DG) mimic glucose but cannot undergo further metabolism, effectively inhibiting HK2 activity (130). Preclinical studies have shown that 2-DG induces apoptosis and enhances the sensitivity of cancer cells to chemotherapy and radiotherapy (116). However, its clinical application is limited by toxicity and lack of specificity. PKM2 controls the final step of glycolysis, converting phosphoenolpyruvate to pyruvate. PKM2 exists in both active tetrameric and less active dimeric forms, with cancer cells favoring the dimeric form to promote anabolic processes. Small-molecule activators like TEPP-46 and DASA-58 stabilize the tetrameric form, enhancing pyruvate production and reducing lactate formation (117, 118). This shift can suppress tumor growth and induce apoptosis.

Cancer cells can compensate for inhibited glycolysis by increasing reliance on mitochondrial OXPHOS. Targeting OXPHOS can disrupt this adaptive mechanism: 1. Complex I Inhibitors: Metformin and phenformin inhibit mitochondrial complex I, reducing ATP production and increasing ROS generation (120, 121). Phenformin has shown greater antitumor activity than metformin in preclinical models of NSCLC but with a higher risk of lactic acidosis (131). 2. ATP Synthase Inhibitors: Oligomycin and its derivatives inhibit ATP synthase, leading to decreased ATP production (122). While effective *in vitro*, their clinical use is limited due to toxicity in normal cells. 3. B-cell lymphoma-2/-xL (Bcl-2/Bcl-xL) Inhibitors: Agents like venetoclax target anti-apoptotic proteins located on the mitochondrial membrane, promoting apoptosis in cancer cells (123). Combining these with OXPHOS inhibitors can enhance cancer cell death.

Combining glycolysis inhibitors with OXPHOS inhibitors may prevent cancer cells from switching energy sources and enhance therapeutic efficacy. Simultaneous targeting of HK2 and complex I can induce energetic crisis in cancer cells. For example, combining 2-DG with metformin has shown synergistic effects in reducing NSCLC cell viability (132). Inhibiting key enzymes in both pathways limits the ability of cancer cells to adapt metabolically. This approach can lead to increased ROS production, DNA damage, and activation of cell death pathways.

### 5.2 Targeting lipid metabolism

Lipid metabolism plays a crucial role in the growth and survival of NSCLC cells, as previously discussed. Targeting key enzymes involved in lipid synthesis and desaturation presents a promising therapeutic strategy. FASN and SCD1 are two pivotal enzymes in lipid metabolism that have gained significant attention as potential targets for cancer therapy.

FASN is an essential enzyme responsible for the *de novo* synthesis of long-chain fatty acids from acetyl-CoA and malonyl-CoA precursors. Overexpression of FASN has been observed in various cancers, including NSCLC, and is associated with poor prognosis and aggressive tumor behavior (133). By promoting lipid synthesis, FASN supports membrane biogenesis, energy storage, and the production of lipid signaling molecules that facilitate tumor growth and metastasis (134). TVB-2640 is a first-in-class, orally bioavailable, small-molecule inhibitor of FASN that has entered clinical trials. It selectively inhibits the enzymatic activity of FASN, leading to reduced fatty acid synthesis and accumulation of malonyl-CoA, which can induce apoptosis and inhibit tumor growth (124). In preclinical studies, TVB-2640 demonstrated significant antitumor activity in NSCLC models, both as a monotherapy and in combination with other agents (125). Clinical trials are currently evaluating the safety and efficacy of TVB-2640 in patients with advanced solid tumors, including NSCLC (124). Preliminary results have shown that TVB-2640 is well-tolerated and exhibits antitumor activity, especially when combined with other treatments such as chemotherapy or targeted therapies. These findings suggest that inhibiting FASN can disrupt lipid homeostasis in cancer cells, leading to growth inhibition and enhanced sensitivity to other anticancer agents.

SCD1 is a rate-limiting enzyme in the synthesis of monounsaturated fatty acids (MUFAs) from saturated fatty acids (SFAs). MUFAs are critical components of cellular membranes and play a role in lipid signaling and energy storage (126). Overexpression of SCD1 has been linked to increased proliferation, survival, and chemoresistance in NSCLC cells (135). Targeting SCD1 disrupts the balance of saturated and unsaturated fatty acids, affecting membrane fluidity and function. Inhibition of SCD1 leads to the accumulation of SFAs, which can cause ER stress and activate the unfolded protein response (UPR) (136). Prolonged ER stress can trigger apoptosis in cancer cells. Additionally, decreased levels of MUFAs impair membrane synthesis and the formation of lipid rafts, which are essential for signal transduction and the activation of oncogenic pathways. Several small-molecule inhibitors of SCD1 have been developed and shown to exhibit antitumor activity in preclinical models of NSCLC. For example, A939572 and MF-438 are potent SCD1 inhibitors that have demonstrated the ability to reduce tumor cell proliferation and induce apoptosis (126, 127). In combination with other treatments, such as chemotherapy or targeted therapies, SCD1 inhibitors may enhance therapeutic efficacy by sensitizing cancer cells to these agents. While targeting lipid metabolism shows promise, clinical outcomes have been variable. Some studies suggest that inhibiting FAO may impair T cell function and exacerbate inflammation, raising concerns about off-target effects. Moreover, conflicting evidence exists regarding the dependency of certain NSCLC subtypes on FAO versus lipogenesis, underscoring the need for subtype-specific therapeutic strategies.

### 5.3 Targeting amino acid metabolism

Amino acid metabolism plays a pivotal role in the growth and survival of NSCLC cells. Targeting key enzymes and pathways involved in amino acid utilization presents a promising therapeutic strategy. Glutaminase (GLS) inhibitors, such as CB-839 (telaglenastat), and inhibitors of serine and glycine synthesis pathways have shown potential in impairing tumor growth by disrupting critical metabolic processes (128). Glutamine is an essential nutrient for rapidly proliferating cancer cells, serving as a carbon and nitrogen source for nucleotide and amino acid synthesis, as well as maintaining redox balance through glutathione production. GLS catalyzes the conversion of glutamine to glutamate, a key step in glutamine metabolism. Overexpression of GLS has been observed in NSCLC and is associated with increased tumor aggressiveness (137). CB-839 is an orally bioavailable, selective GLS inhibitor that has demonstrated antitumor activity in preclinical models of NSCLC by blocking glutamine utilization (138). By inhibiting GLS, CB-839 reduces the production of glutamate and downstream metabolites, leading to impaired nucleotide synthesis, decreased glutathione levels, and increased oxidative stress (139). This can result in cancer cell death and reduced tumor growth.

Serine and glycine are non-essential amino acids integral to one-carbon metabolism, which is crucial for nucleotide synthesis, methylation reactions, and maintaining redox balance (54). NSCLC cells often upregulate enzymes involved in the serine-glycine synthesis pathway to meet the increased demands of rapid proliferation. PHGDH catalyzes the first step in the *de novo* serine synthesis pathway. Overexpression of PHGDH has been observed in NSCLC and is associated with enhanced tumor growth (140). Inhibitors targeting PHGDH can disrupt serine production, impair nucleotide biosynthesis, and induce cell cycle arrest. Novel PHGDH inhibitors have shown efficacy in preclinical models, reducing tumor growth and enhancing sensitivity to chemotherapy (141). SHMT converts serine to glycine, contributing to one-carbon units necessary for thymidine and purine synthesis (142). Inhibiting SHMT disrupts DNA synthesis and can induce apoptosis in cancer cells. Agents targeting SHMT have demonstrated antitumor activity by inducing DNA damage and impairing cell proliferation (143).

Proline metabolism is another pathway exploited by NSCLC cells to support tumor growth and metastasis (144). Proline biosynthesis and degradation are linked to energy production and redox balance. Inhibiting key enzymes such as pyrroline-5-carboxylate reductase (PYCR) can disrupt proline metabolism, leading to increased oxidative stress and reduced tumor growth (145). Targeting proline metabolism may also impair the survival of cancer stem cells, which are often resistant to conventional therapies (146).

BCAAs-leucine, isoleucine, and valine-are essential amino acids involved in protein synthesis and signaling pathways that regulate cell growth (147). NSCLC cells may exhibit increased uptake and catabolism of BCAAs to fuel the TCA cycle and provide nitrogen for nucleotide and amino acid synthesis (148). BCAT1 catalyzes the first step in BCAA catabolism. Overexpression of BCAT1 has been associated with tumor aggressiveness and poor prognosis in NSCLC (149). Inhibiting BCAT1 can impair BCAA metabolism, suppress tumor growth, and reduce cancer cell proliferation. To support increased amino acid demands, NSCLC cells often upregulate amino acid transporters (150). Targeting these transporters can reduce the uptake of critical nutrients: ASCT2 is a glutamine transporter overexpressed in many cancers (151). Inhibiting ASCT2 can decrease glutamine uptake, leading to metabolic stress and sensitizing cancer cells to chemotherapy (152). LAT1 transports large neutral amino acids, including leucine (153). Targeting LAT1 can disrupt mTOR signaling pathways, reduce protein synthesis, and inhibit tumor growth (65). Similarly, targeting amino acid metabolism is not without limitations. For instance, while LAT1 inhibitors show antitumor potential, they may also impact immune cell metabolism or lead to resistance via transporter redundancy. Furthermore, some clinical trials targeting amino acid pathways have failed to show significant benefits, highlighting the complexity and redundancy of metabolic networks in cancer.

### 5.4 Combination therapies and immunometabolism

Combining metabolic inhibitors with immunotherapies has emerged as a promising strategy to enhance antitumor immune responses in NSCLC (154). Modulating tumor metabolism can improve the function of immune cells within the TME, representing a synergistic approach to NSCLC treatment. Tumor cells often create a metabolically hostile environment for immune cells by consuming large amounts of glucose and amino acids, leading to nutrient deprivation for tumor-infiltrating lymphocytes (TILs) (155). Additionally, tumor cells produce immunosuppressive metabolites like lactate and adenosine, which inhibit immune cell function (156). By targeting tumor metabolism, it is possible to alleviate these immunosuppressive conditions and enhance the efficacy of immunotherapies. Inhibiting glycolysis in tumor cells can reduce glucose competition, making it more available for effector T cells that rely on glycolysis for their function (157). Glycolytic inhibitors, such as 2-DG, may improve T-cell activity when combined with immune checkpoint inhibitors (116). IDO is an enzyme overexpressed in some tumors that depletes tryptophan and produces immunosuppressive metabolites (158). IDO inhibitors can restore tryptophan levels and enhance T-cell proliferation. Combining IDO inhibitors with PD-1/PD-L1 blockade has shown synergistic antitumor effects in preclinical models (159). As discussed previously, GLS inhibitors like CB-839 can reduce glutamine availability for tumor cells (128). Since glutamine is less critical for T-cell function than for tumor cells, GLS inhibition may preferentially affect cancer cells and improve immune responses.

Modulating the metabolism of immune cells themselves can also enhance antitumor immunity. The mTOR pathway regulates T-cell metabolism and function (160). Activating mTOR can promote T-cell glycolysis and effector functions. Agents that enhance mTOR signaling in T cells may boost their antitumor activity. AMPK activation can improve the metabolic fitness of T cells, enhancing their survival and function in the nutrient-deprived TME (161).

Combining metabolic inhibitors with immunotherapies aims to create a more favorable metabolic environment for immune cells while directly targeting tumor metabolism. Targeting metabolic checkpoints in tumor cells can sensitize them to immune-mediated killing. For example, inhibiting LDHA reduces lactate production, alleviating acid-mediated immunosuppression (119). Combining immune checkpoint inhibitors (e.g., anti-PD-1/PD-L1 antibodies) with metabolic modulators can enhance T-cell infiltration and activity. Clinical trials are exploring such combinations in NSCLC patients. Epacadostat, an IDO inhibitor, has been evaluated in combination with pembrolizumab (anti-PD-1) in clinical trials, showing promising results in some cancer types (162). However, results have been mixed, and further studies are needed to determine efficacy in NSCLC. Metformin, a complex I inhibitor, has immunomodulatory effects and may enhance responses to immunotherapy (132). Retrospective studies suggest that NSCLC patients taking metformin may have improved outcomes with immune checkpoint inhibitors.

### 5.5 Personalized metabolic therapies

The heterogeneity of metabolic profiles among NSCLC tumors underscores the need for personalized metabolic therapies (163). Metabolic profiling of tumors can identify patient-specific metabolic dependencies, enabling tailored treatment strategies that target the unique metabolic vulnerabilities of each tumor (164). Advanced technologies such as metabolomics, genomics, transcriptomics, and proteomics allow for comprehensive metabolic profiling of tumors. High-throughput techniques can analyze metabolic enzyme expression levels, metabolite concentrations, and metabolic fluxes within cancer cells (165). These high-throughput techniques include: 1. Mass Spectrometry (MS) and Nuclear Magnetic Resonance (NMR) Spectroscopy: These techniques enable the identification and quantification of a wide range of metabolites in tumor samples. 2. Positron Emission Tomography (PET) Imaging: Metabolic imaging using tracers like 18F-fluorodeoxyglucose (FDG) can assess glucose uptake in tumors, providing insights into glycolytic activity. 3. Gene Expression Profiling: Analyzing the expression of genes involved in metabolic pathways can reveal overactive or dysregulated metabolic enzymes.

## 6 Challenges and future directions

Cancer cells’ ability to adapt metabolically poses a significant challenge for metabolic therapies. Their metabolic plasticity enables them to switch between different energy sources and metabolic pathways, which can render single-agent treatments less effective. Future research should focus on understanding these resistance mechanisms and developing strategies to prevent or overcome them. Ensuring the safety and selectivity of metabolic inhibitors is also critical; these agents must selectively target cancer cells without harming normal tissues. Strategies to achieve this include exploiting cancer-specific metabolic pathways or employing delivery systems that preferentially target tumor cells.

Integrating metabolic biomarkers into clinical practice is essential for personalizing treatment and improving outcomes. Standardizing these biomarkers and incorporating them into clinical workflows require close collaboration between researchers and clinicians. Validation in large, diverse patient cohorts is necessary to ensure their reliability and effectiveness in clinical settings. Advancing the field further necessitates interdisciplinary collaboration among oncologists, biochemists, pharmacologists, and computational biologists. Integrating diverse expertise will accelerate the translation of metabolic discoveries into effective therapies, ultimately enhancing treatment strategies for NSCLC.

## 7 Conclusion

The metabolic reprogramming of NSCLC cells extends far beyond the Warburg effect, encompassing alterations in lipid and amino acid metabolism and dynamic interactions with the TME. These metabolic adaptations are not merely bystanders but are integral to tumor growth, survival, and therapeutic resistance. By leveraging emerging technologies and a deeper understanding of metabolic heterogeneity, we can identify novel vulnerabilities in NSCLC. Therapeutic strategies that target these metabolic dependencies offer promising avenues for improving patient outcomes. Combining metabolic inhibitors with existing treatments, such as immunotherapies and targeted therapies, may enhance efficacy and overcome resistance. Personalized metabolic therapies, guided by metabolic profiling, represent the next frontier in precision oncology. As we continue to unravel the complexities of NSCLC metabolism, we move closer to realizing the full potential of metabolic targeting in cancer therapy. The integration of cutting-edge research with clinical practice holds the promise of transforming NSCLC management and improving the lives of patients worldwide.
